# Histone Methyltransferases MES-4 and MET-1 Promote Meiotic Checkpoint Activation in *Caenorhabditis elegans*

**DOI:** 10.1371/journal.pgen.1003089

**Published:** 2012-11-15

**Authors:** Piero Lamelza, Needhi Bhalla

**Affiliations:** Department of Molecular, Cell, and Developmental Biology, University of California Santa Cruz, Santa Cruz, California, United States of America; University of California Davis, United States of America

## Abstract

Chromosomes that fail to synapse during meiosis become enriched for chromatin marks associated with heterochromatin assembly. This response, called meiotic silencing of unsynapsed or unpaired chromatin (MSUC), is conserved from fungi to mammals. In *Caenorhabditis elegans*, unsynapsed chromosomes also activate a meiotic checkpoint that monitors synapsis. The synapsis checkpoint signal is dependent on *cis*-acting loci called Pairing Centers (PCs). How PCs signal to activate the synapsis checkpoint is currently unknown. We show that a chromosomal duplication with PC activity is sufficient to activate the synapsis checkpoint and that it undergoes heterochromatin assembly less readily than a duplication of a non-PC region, suggesting that the chromatin state of these loci is important for checkpoint function. Consistent with this hypothesis, MES-4 and MET-1, chromatin-modifying enzymes associated with transcriptional activity, are required for the synapsis checkpoint. In addition, a duplication with PC activity undergoes heterochromatin assembly when *mes-4* activity is reduced. MES-4 function is required specifically for the *X* chromosome, while MES-4 and MET-1 act redundantly to monitor autosomal synapsis. We propose that MES-4 and MET-1 antagonize heterochromatin assembly at PCs of unsynapsed chromosomes by promoting a transcriptionally permissive chromatin environment that is required for meiotic checkpoint function. Moreover, we suggest that different genetic requirements to monitor the behavior of sex chromosomes and autosomes allow for the lone unsynapsed *X* present in male germlines to be shielded from inappropriate checkpoint activation.

## Author Summary

Sexual reproduction relies on meiosis. This specialized cell division generates gametes, such as sperm and eggs, with a single copy of the genome so that fertilization restores diploidy. During meiosis, homologous chromosomes undergo synapsis, in which they assemble a proteinaceous structure called the synaptonemal complex to promote proper chromosome segregation. In *C. elegans*, a checkpoint monitors synapsis and removes nuclei that have unsynapsed chromosomes by activating programmed cell death or apoptosis. Activation of this checkpoint coincides with, but is not dependent on, the assembly of heterochromatin on unsynapsed chromosomes. The acquisition of heterochromatic marks is a conserved response to asynapsis. A specific portion of each chromosome, called a Pairing Center (PC), is required for this checkpoint; but how PCs send a signal when chromosomes are unsynapsed is unknown. We present evidence that suggests that unsynapsed PCs need to maintain a transcriptionally open chromatin environment, even as the rest of the chromosome undergoes heterochromatin assembly, to activate the checkpoint. Furthermore, we demonstrate that sex chromosomes have different genetic requirements than autosomes to monitor synapsis, providing a potential explanation for observed sexual dimorphism in synapsis checkpoint activation.

## Introduction

Meiosis is the specialized cell division in which a diploid cell gives rise to haploid gametes, such as eggs and sperm. To halve the chromosome complement, meiosis is composed of two divisions without an intervening S phase: meiosis I, in which homologous chromosomes are segregated, and meiosis II, in which sister chromatids are segregated. To ensure that chromosomes segregate properly in meiosis and produce gametes with the correct number of chromosomes, homologous chromosomes undergo meiosis-specific events to generate a linkage, or chiasma, that enables proper biorientation on the meiotic spindle. Homologous chromosomes identify their unique partner to pair, stabilize this pairing by assembling a proteinaceous structure called the synaptonemal complex (SC) between homologs and undergo homologous recombination in the context of the SC. Defects in SC formation prevent or severely reduce homologous recombination [1] and therefore can produce gametes with an incorrect chromosome complement. Fertilization of these defective gametes can result in embryos that are inviable or have serious developmental disorders, such as Down and Klinefelter's syndrome in humans. It is estimated that 30% of miscarriages are the product of meiotic chromosome missegregation [2].

Meiotic checkpoints maintain genomic integrity by monitoring events, such as synapsis and recombination, to make sure that they occur properly, in a timely manner and in the appropriate order. If meiotic events are disrupted, these surveillance mechanisms delay cell cycle progression to enable cells to correct errors or activate apoptosis to remove defective cells [3]. During oogenesis in *Caenorhabditis elegans*, a checkpoint monitors synapsis independent of recombination and activates apoptosis to remove nuclei with unsynapsed chromosomes to prevent the production of aneuploid oocytes [4]. The checkpoint signal requires the presence of unsynapsed Pairing Centers (PCs) [4], *cis*–acting sites near one end of each chromosome that promote pairing and synapsis [5]–[7]. How PCs activate the synapsis checkpoint is currently unknown.

The presence of unpaired or unsynapsed chromosomes results in meiotic silencing of unsynapsed chromatin (MSUC), a response conserved from fungi to mammals [8]–[10]. During MSUC, unpaired or unsynapsed chromosomes become decorated with chromatin modifications associated with transcriptional silencing or heterochromatin formation [11]. In mammals, MSUC shares features with meiotic sex chromosome inactivation (MSCI), the process by which the partially synapsed sex chromosomes (the X and Y) are transcriptionally silenced during spermatogenesis [11]. Indeed, the presence of unsynapsed autosomes can deplete factors required for MSCI [12] and induce apoptosis as a result of inappropriate gene expression from the Y chromosome [13], raising the possibility that competition for these resources may act as a reporter for defects in synapsis, at least during spermatogenesis. During oogenesis, MSUC-induced transcriptional silencing of chromosomal loci essential for meiosis has been proposed to induce apoptosis [14].

In *C. elegans*, both MSUC and MSCI result in decoration of unsynapsed chromosomes with dimethylated lysine 9 on histone H3 (H3K9me2), a chromatin modification consistent with transcriptional silencing [8]. However, MSCI and MSUC occur by distinct mechanisms in this organism [15]. During spermatogenesis in *XO* males, *X* chromosomes remain unsynapsed and undergo MSCI [8], which prevents meiotic checkpoint activation [16]. MSCI is dependent on a conserved SET domain histone methyltransferase, MET-2, as loss of this protein reduces H3K9me2 accumulation on the single *X* [17], increases the transcriptional activity of the single *X* and activates a DNA damage checkpoint in response to defects in recombination [15]. Loss of MET-2 during oogenesis in *XX* hermaphrodites also affects the chromatin state of unsynapsed chromosomes, in that they are no longer enriched with H3K9me2 [17], but there is no corresponding increase in transcriptional activity or checkpoint activation [15], indicating that MSUC and MSCI are not equivalent processes. Furthermore, MSUC appears to be the consequence of several pathways [17]–[19], one of which is not involved in MSCI [15].

In *C. elegans*, *mes-4* and *met-1* encode well-characterized histone methyltransferases associated with active transcription, specifically the catalysis of H3K36me [20]. MES-4 is a critical regulator of germline development and immortality [21] and primarily binds transcriptionally active autosomes [22]; in *mes-2*, *mes-3* and *mes-6* mutants, MES-4 mislocalizes along the *X* chromosome [22], possibly as a result of the inappropriate upregulation of *X*-linked genes in these mutants [20]. MET-1 is responsible for RNA Polymerase II dependent H3K36me3 deposition [20], most likely by the mechanism described for its ortholog, budding yeast Set2. Yeast Set2 associates with Pol II during its elongation phase and catalyzes H3K36me in the body of genes to recruit transcriptional repressors that prevent cryptic transcription initiation [23]–[25]. MET-1 also contributes to *C. elegans* vulval development [26].

Given the requirement for *cis*-acting loci in synapsis checkpoint activation, we wondered if the chromatin state of PCs was important for checkpoint function. Specifically, if unsynapsed chromosomes appear to undergo heterochromatin assembly, do PCs behave differently to contribute to checkpoint activation? To answer this question, we investigated the importance of chromatin modifying enzymes during synapsis checkpoint activation. We show that unsynapsed chromosomes are decorated with H3K9me2 whether they activate the DNA damage checkpoint or the synapsis checkpoint, indicating that heterochromatin assembly is a general response to asynapsis. Indeed, these checkpoints appear to be dispensable for H3K9me2 enrichment on unsynapsed chromosomes. However, a chromosomal duplication that harbors PC activity and is sufficient for synapsis checkpoint activation undergoes heterochromatin assembly less readily than a chromosomal duplication of a non-PC region. We also report that MES-4 and MET-1 are required for the synapsis checkpoint. Consistent with these enzymes acting at PCs, a duplication with PC activity becomes enriched with H3K9me2 when *mes-4* activity is reduced. Taken together, our data suggest that these chromatin-modifying enzymes antagonize heterochromatin assembly at PCs to promote checkpoint activation. Therefore, chromatin state, and potentially transcriptional activity, at these *cis*-acting sites correlate with checkpoint signaling. Moreover, *C. elegans* sex chromosomes exhibit different genetic requirements than autosomes to activate the synapsis checkpoint: MES-4 is specifically required to monitor synapsis of *X* chromosomes while MES-4 and MET-1 are redundant for synapsis checkpoint activation when autosomes are unsynapsed. These results may explain why the single X chromosome in males does not activate the synapsis checkpoint despite being unsynapsed.

## Results

### Heterochromatin assembly is a general response to unsynapsed chromosomes

We wanted to determine whether chromosomes that activate the synapsis checkpoint also become enriched for H3K9me2. Most observations of heterochromatin assembly on unpaired or unsynapsed chromosomes have been in situations in which chromosomal duplications are present in meiotic nuclei or unpaired chromosomes activate a meiotic checkpoint that monitors recombination defects (also known as the DNA damage checkpoint) [8], [15]–[19]. We tested whether unsynapsed chromosomes became enriched for H3K9me2 in a genotype in which only the synapsis checkpoint is activated.

We have shown that a single pair of unsynapsed chromosomes can robustly activate either the synapsis checkpoint or the DNA damage checkpoint, depending on whether the unsynapsed chromosomes include active PCs [4]. *meDf2* is a deficiency that removes up to 2 Mb of the left end of the *X* chromosome and the *X* chromosome Pairing Center (PC) [27]. Animals homozygous for *meDf2* exhibit unsynapsed *X* chromosomes in almost all meiotic nuclei [5]. Unsynapsed *X* chromosomes in *meDf2* homozygotes do not have an active PC and therefore activate the DNA damage checkpoint and not the synapsis checkpoint [4]. Animals heterozygous for *meDf2* exhibit unsynapsed *X* chromosomes in 60% of meiotic nuclei [5]. Since the synapsis checkpoint requires an active Pairing Center (PC), meiotic nuclei with unsynapsed chromosomes in *meDf2* heterozygotes activate the synapsis checkpoint [4]. However, for reasons that are not known, the DNA damage checkpoint is not activated in *meDf2* heterozygotes.

**Figure 1:**
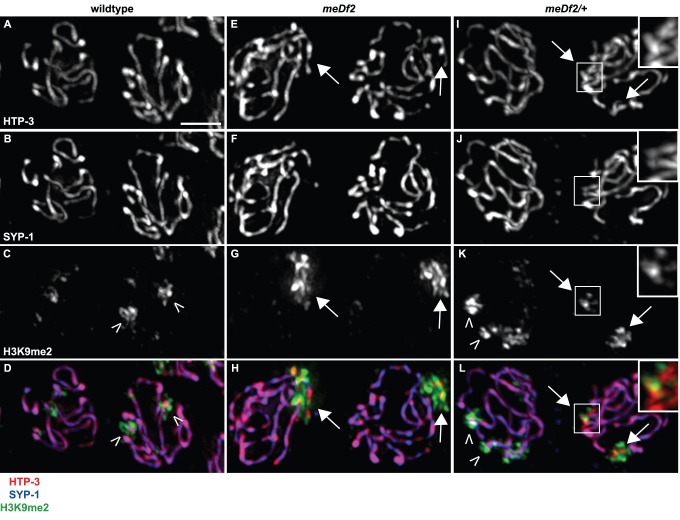
H3K9me2 is enriched on unsynapsed chromosomes when either the DNA damage checkpoint or the synapsis checkpoint is activated. Indirect immunofluorescence was performed on meiotic nuclei in wildtype, *meDf2* homozygotes and *meDf2* heterozygotes using antibodies against the axial element component HTP-3, the central element component SYP-1 and H3K9me2. Wildtype meiotic nuclei exhibit colocalization of HTP-3 and SYP-1 (A, B and D), and have areas of H3K9me2 enrichment (carets) and depletion (C and D). Meiotic nuclei in both *meDf2* homozygotes and *meDf2* heterozygotes have chromosomes with HTP-3 but not SYP-1 (arrows in E, F, H, I, J and L, magnified insets in I, J and L). These are unsynapsed *X* chromosomes and are highly decorated with H3K9me2 (G, H, K and L, magnified insets in K and L). Other regions of H3K9me2 enrichment are also observed in nuclei with synapsed chromosomes in *meDf2* heterozygotes (carets in K and L). Scale bar represents 4 microns.

We directly assessed synapsis by performing indirect immunofluorescence against the synaptonemal complex components, HTP-3 [5] and SYP-1 [28], in meiotic nuclei. HTP-3 is an axial element component that loads onto chromosomes axes prior to and independent of synapsis [5]. SYP-1 is a central element component that polymerizes between homologous chromosomes as they synapse [28]. In wildtype mid-pachytene nuclei, we observed colocalization of HTP-3 and SYP-1 (Figure 1A, 1B and 1D), consistent with complete synapsis, and chromosomal regions where H3K9me2 is depleted or enriched (Figure 1C and 1D, carets indicate regions of enrichment), as previously reported [17]. In meiotic nuclei in *meDf2* homozygotes, we observed stretches of HTP-3 devoid of SYP-1 (arrows in Figure 1E and 1H): these are unsynapsed *X* chromosomes [5] and they are the primary source of H3K9me2 signal in these nuclei (Figure 1G). In *meDf2* heterozygotes, some nuclei completed synapsis (nucleus on left in Figure 1I–1L) while other nuclei exhibited unsynapsed *X* chromosomes (arrows in Figure 1I, 1K and 1L). Nuclei with synapsed *X* chromosomes exhibited wildtype localization of H3K9me2, while nuclei with unsynapsed *X* chromosomes had H3K9me2 limited to regions of asynapsis (arrows in Figure 1K and 1L). Therefore, enrichment of H3K9me2 on unsynapsed chromosomes occurs whether the DNA damage or synapsis checkpoint is activated. Furthermore, heterochromatin assembly on unsynapsed chromosomes is an event each meiotic nucleus undertakes independently; neighboring nuclei can exhibit different H3K9me2 patterns depending on their state of synapsis (Figure 1I–1L).

### A duplication that contains PC activity and activates the synapsis checkpoint often fails to undergo heterochromatin assembly

**Figure 2:**
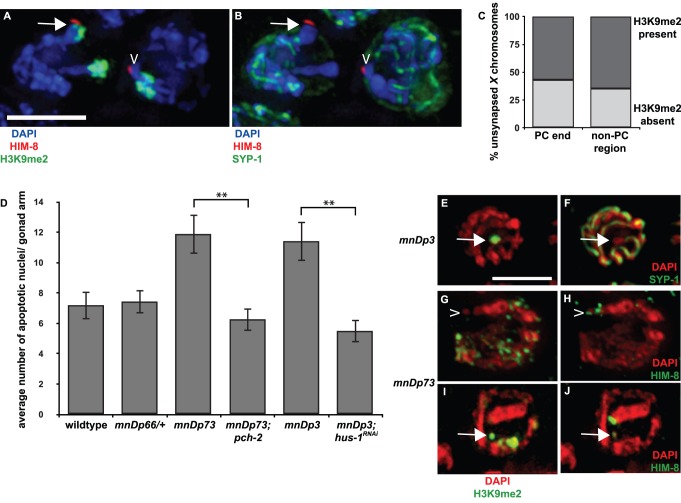
A chromosomal duplication with Pairing Center activity often does not undergo heterochromatin assembly. A and B. In *meDf2/+* hermaphrodites, it is difficult to determine if PCs of unsynapsed *X* chromosomes become enriched with H3K9me2. Indirect immunofluorescence was performed on meiotic nuclei in *meDf2* heterozygotes using antibodies against the *X* chromosome PC protein HIM-8, the central element component SYP-1 and H3K9me2. The nucleus on the left contains an unsynapsed *X* chromosome, as identified by HIM-8, an enrichment of H3K9me2 (A) and the absence of SYP-1 (B); the PC appears enriched with H3K9me2 (arrow in A). The nucleus on the right also contains an unsynapsed *X* chromosome, as indicated by the absence of SYP-1 in B, and the PC of this chromosome does not exhibit robust H3k9me2 (caret in A). C. Quantification of the percentage of unsynapsed *X* chromosomes in *meDf2* heterozygotes that were enriched for H3K9me2 at their PC and non-PC regions. D. A chromosomal duplication with Pairing Center activity (*mnDp73*) activates the synapsis checkpoint. A duplication that does not have Pairing Center activity (*mnDp66/+*) fails to activate the synapsis checkpoint and a duplication of the non-PC portion of the *X* chromosome (*mnDp3*) activates the DNA damage checkpoint. A ** indicates a p value of <0.0001. Error bars indicate 2XSEM. E–J. Indirect immunofluorescence was performed on meiotic nuclei from hermaphrodites carrying *mnDp3* (non-PC end of the *X* chromosome) and *mnDp73* (PC end of the *X* chromosome) with antibodies against H3K9me2, SYP-1 or HIM-8. *mnDp3* is unsynapsed (arrow in F) and highly decorated with H3K9me2 (arrow in E). This was observed in 100% of meiotic nuclei (23 of 23) in which *mnDp3* could unambiguously be identified as unsynapsed. *mnDp73* is identified as a free DAPI staining body that recruits HIM-8 (caret in H and arrow in J) but that is often not enriched with H3K9me2 (caret in G). This was observed in 13 out of 17 in which *mnDp73* could unambiguously be identified by HIM-8 recruitment. In the remaining four nuclei in which *mnDp73* was identified by HIM-8 binding (arrow in J), the duplication was enriched for H3K9me2 (arrow in I) but was not the primary signal of H3K9me2 in the nucleus. Scale bar represents 4 microns.

We wanted to more closely examine the relationship between H3K9me2 and PCs. We analyzed the localization of H3K9me2 and the *X* chromosome PC protein HIM-8 [7] in meiotic nuclei with unsynapsed *X* chromosomes in *meDf2* heterozygotes. In nuclei with unsynapsed *X* chromosomes, as identified by the enrichment of H3K9me2 (Figure 2A) and absence of SYP-1 (Figure 2B), we sometimes observed a reduction in H3K9me2 in the vicinity of HIM-8 staining (caret in Figure 2A) but we also observed robust H3K9me2 adjacent to HIM-8 (arrow in Figure 2A and Figure S1). We quantified the frequency of H3K9me2 enrichment at the PC end and non-PC end of unsynapsed chromosomes in *meDf2* heterozygotes (Figure 2C). Of meiotic nuclei with unsynapsed X chromosomes (n = 320), 43% did not exhibit enriched H3K9me2 at the PC end (Figure 2C). However, this was only slightly higher than the percentage of unsynapsed X chromosomes that did not exhibit H3K9me2 enrichment at a non-PC region (35%) (Figure 2C), making it difficult for us to draw any firm conclusions about the chromatin state of unsynapsed PCs in synapsis checkpoint activating strains.

We decided to take advantage of chromosomal duplications, such as *mnDp73*, that have demonstrated PC activity: they are able to recombine with full-length *X* chromosomes and disrupt their proper segregation in hermaphrodites [29]. To determine if *mnDp73*, which is a free duplication, could recapitulate all functions of PCs, we addressed whether it activated the synapsis checkpoint. When we assayed germline apoptosis in hermaphrodites carrying *mnDp73*, we observed an increase in apoptosis above the physiological levels of apoptosis observed in wildtype. This increase in apoptosis is dependent on *pch-2* (Figure 2D and Table 1), a component of the synapsis checkpoint [4]. This is in contrast to a duplication of the PC end of the *X* chromosome that lacks PC activity (*mnDp66*, which is attached to Chromosome I) [29]. Despite being unsynapsed (data not shown), animals that are heterozygous for *mnDp66* did not exhibit elevated apoptosis (Figure 2D and Table 1), indicating that synapsis checkpoint activation correlates with other measures of PC activity. Animals carrying a free duplication of the non-PC end of the X chromosome, *mnDp3* [30], also exhibited elevated germline apoptosis but this elevation was dependent on the DNA damage checkpoint, as knock down of the DNA damage checkpoint component *hus-1* [31] by RNA interference in animals carrying *mnDp3* reduced apoptosis to wildtype levels (Figure 2D and Table 1). We have previously shown that mutation or inactivation of *pch-2* or *hus-1* by RNAi in an otherwise wildtype genetic background has no effect on physiological apoptosis [4].

**Table 1:** Duplications used in this study.

Duplication	checkpoint activation?	Enriched for H3K9me2?
*mnDp66*	no	N/A
*mnDp73* (PC end of *X*)	synapsis checkpoint activated	24% (4/17 nuclei scored)
*mnDp3* (non-PC end of *X*)	DNA damage checkpoint activated	100% (23/23 nuclei scored)

Having verified that the presence of *mnDp73* was sufficient for synapsis checkpoint activation, we assessed if it, like other duplications characterized, underwent heterochromatin assembly. We performed indirect immunofluorescence on animals that contained either *mnDp73* (Figure 2G and 2I) or *mnDp3* (Figure 2E) [30] with an antibody against H3K9me2. In germlines of hermaphrodites carrying *mnDp3*, we identified free DAPI staining bodies in mid-pachytene nuclei that did not load SYP-1 (Figure 2F) and observed what proportion of these stained with H3K9me2. Of 23 nuclei in which we could unambiguously identify unsynapsed DAPI staining bodies, all 23 exhibited robust H3K9me2; the unsynapsed duplication was the primary source of H3K9me2 signal in these nuclei (Figure 2E and Table 1). We noticed that unambiguously unsynapsed DAPI staining bodies in the meiotic nuclei of *mnDp3* bearing animals were rare. Often we could not observe an unsynapsed DAPI-staining body in most meiotic nuclei consisting of a single H3K9me2 body characteristic of nuclei containing a chromosomal duplication (arrows in Figure S2). We determined the percentage of nuclei that included a single H3K9me2 body that was also unsynapsed and found that only 38% (23 of 60) of H3K9me2 enriched duplications were unambiguously unsynapsed, suggesting that heterochromatin assembly is a response to the unpaired status of chromosomes as opposed to the unsynapsed status of chromosomes.

**Figure 3:**
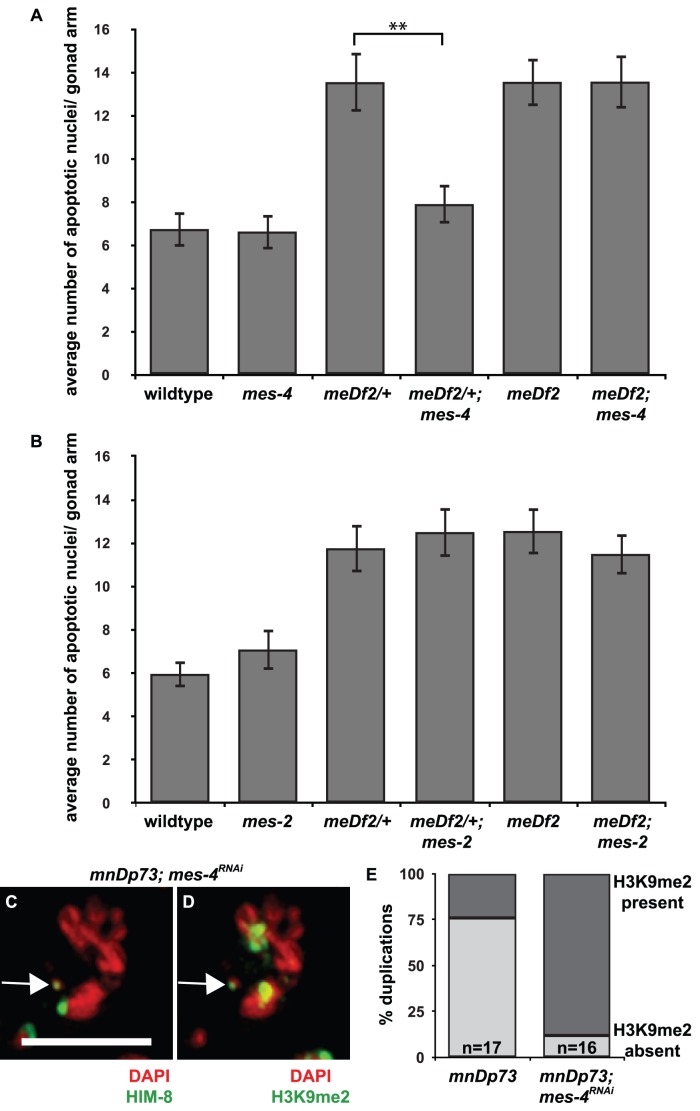
*mes-4* is required for the synapsis checkpoint and prevents heterochromatin assembly on a chromosomal duplication with PC activity. A. Mutation of *mes-4* reduces apoptosis in *meDf2/+* but not in *meDf2* homozygotes. A ** indicates a p value of <0.0001. B. Mutation of *mes-2* does not reduce apoptosis in *meDf2/+* or *meDf2* homozygotes. Error bars indicate 2XSEM. C and D. Indirect immunofluorescence was performed on meiotic nuclei in hermaphrodites carrying *mnDp73* in which *mes-4* was knocked down by RNAi with antibodies against H3K9me2 and HIM-8. *mnDp73* is identified as a free duplication bound by HIM-8 (arrow in C). In 14 out of 16 in which the duplication could be identified, *mnDp73* is enriched with H3K9me2 (arrow in D). Scale bar represents 4 microns. E. Quantification of the percentage of duplications that were enriched with H3K9me2 in *mnDp73* bearing hermaphrodites and *mnDp73* bearing hermaphrodites in which *mes-4* was inactivated by RNAi.

Because of the substantially smaller size of *mnDp73* (see Figure 2G–2J, [29]), we identified the duplication as bound by HIM-8 (caret in Figure 2H and arrow in 2J) and observed what proportion of these were enriched with H3K9me2; this accounts for the smaller sample size we report for this duplication. In contrast to *mnDp3*, *mnDp73* appeared as a free DAPI staining body without H3K9me2 in a majority (13 out of 17) of meiotic nuclei in which the duplication could be identified (caret in Figure 2G and Figure 3E). Four of the seventeen meiotic nuclei in which *mnDp73* could be identified were enriched for H3K9me2 (Figure 2I, Figure 3E and Table 1) but this was not the dominant H3K9me2 signal in these meiotic nuclei, as observed when whole chromosomes are unsynapsed (Figure 1G and 1H) or a larger duplication is present (Figure 2E). Thus, our data support a model in which a duplication that contains PC activity and activates the synapsis checkpoint often fails to undergo heterochromatin assembly.

### MES-4 is required for the synapsis checkpoint when *X* chromosomes are unsynapsed and prevents heterochromatin assembly on a duplication with PC activity

To further investigate the potential link between the exclusion of heterochromatin from PCs that we observed and synapsis checkpoint activation, we tested the role of *mes-4* in the synapsis checkpoint. MES-4 is a histone methyltransferase that is required for germline viability and development [32] and methylates lysine 36 of histone H3 (H3K36me), a methyl mark associated with active chromatin [20], [33]. In embryos, it associates specifically with transcriptionally active autosomes [22] and the leftmost tip of the *X*, where the PC is located [33]. We introduced a *mes-4* mutation into *meDf2* heterozygote hermaphrodites (*meDf2/+*). As a result of synapsis checkpoint activation in *meDf2* heterozygotes [4], germline apoptosis is elevated (Figure 3A). When we assayed apoptosis in *mes-4; meDf2/+* double mutants, we observed a reduction in apoptosis compared to *meDf2/+* single mutants (p value <0.0001) (Figure 3A), indicating that *mes-4* is required for the synapsis checkpoint in *meDf2* heterozygotes. We also assayed whether *mes-4* was required for the DNA damage checkpoint by investigating levels of germline apoptosis in *mes-4; meDf2* animals. *meDf2* hermaphrodites activate the DNA damage checkpoint, which also produces increased levels of germline apoptosis [4]. We did not observe a decrease in apoptosis in *mes-4; meDf2* double mutants, when compared to *meDf2* single mutants (Figure 3A), indicating that *mes-4* is not required for the DNA damage checkpoint.

In addition to *mes-4*, *mes-2*, *mes-3* and *mes-6* are required maternally for normal development of the germline [32] and evidence indicates that these four genes collaborate to promote germline development [22], [34]. MES-2 is a SET domain containing protein orthologous to the Drosophila Polycomb group protein Enhancer of Zeste [35]. MES-2, in a complex with MES-3 and MES-6, catalyzes repressive H3K27me to maintain *X* chromosome silencing in the germline [34], [36], [37]. We tested *mes-2* for a role in the synapsis checkpoint by introducing a *mes-2* mutation into *meDf2* heterozygotes. We did not observe a reduction in apoptosis in the double mutant when compared to the *meDf2* heterozygote (p value = 0.35) (Figure 3B). We also did not observe a decrease in apoptosis in *meDf2* homozygotes when *mes-2* function was absent, indicating that *mes-2* is also not required for the DNA damage checkpoint (Figure 3B). Similar results were obtained with *mes-3* mutants (data not shown). Therefore, the role of MES-4 in the synapsis checkpoint is independent of the MES-2, MES-3 and MES-6 complex.

*mes-4* is not required for MSCI in male germlines [22]. We determined whether it was required for heterochromatin assembly on unsynapsed chromosomes in hermaphrodite germlines and observed no reduction in H3K9me2 on unsynapsed chromosomes in either *meDf2* homozygotes or *meDf2* heterozygotes in which *mes-4* had been mutated (data not shown), indicating that deposition of H3K9me2 on unsynapsed chromosomes is not dependent on activating the synapsis checkpoint. We also investigated whether knock down of *mes-4* by RNAi affected heterochromatin assembly on *mnDp73*; efficient knockdown of *mes-4* in hermaphrodite germlines was verified by performing immunofluorescence against MES-4 (data not shown). We once again identified *mnDp73* in mid-pachytene meiotic nuclei as bound by HIM-8 (Figure 3C) and determined its H3K9me2 state. We found that 14 out of 16 meiotic nuclei in which the duplication could be unambiguously identified as being bound by HIM-8 were also enriched for H3K9me2 (Figure 3D and 3E). Thus in the absence of MES-4, the synapsis checkpoint is inactivated and a duplication with PC activity undergoes heterochromatin assembly, suggesting a link between these events.

It has recently been shown that *plk-2* regulates both a delay in meiotic progression in response to asynapsis and the synapsis checkpoint [38]. We observed that *mes-4* abrogated the synapsis checkpoint in *meDf2/+* hermaphrodites (Figure 3A) without affecting the accompanying delay in meiotic progression or any molecular markers that correlate with this delay, i.e. phosphorylation of SUN-1 at residues serine 8 (Figure S3) and serine 12 (Figure S4) [39], leading us to speculate that MES-4 either works downstream or independent of PLK-2 in the synapsis checkpoint pathway [38].

### MES-4 and MET-1 are redundant for the synapsis checkpoint when all chromosomes are unsynapsed

**Figure 4:**
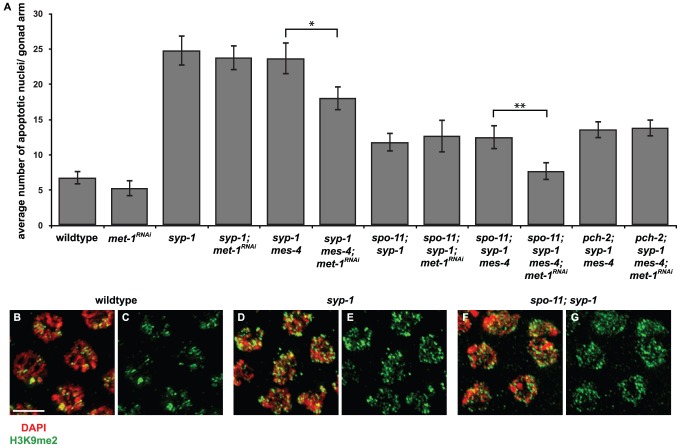
*mes-4* and *met-1* are redundant for synapsis checkpoint activation when all chromosomes are unsynapsed. A. Inactivation of both *mes-4* (by mutation) and *met-1* (by RNA interference) is required to reduce apoptosis in *syp-1* and *spo-11; syp-1* mutants but does not affect apoptosis in *pch-2; syp-1* mutants. A * indicates a p value of <0.05 and a ** indicates a p value of <0.0001. Error bars indicate 2XSEM. B–G. Heterochromatin assembly occurs on unsynapsed chromosomes in *syp-1* and *spo-11; syp-1* mutants. Indirect immunofluorescence was performed on meiotic nuclei in wildtype, *syp-1* and *spo-11; syp-1* double mutants with an antibody against H3K9me2. Wildtype nuclei exhibit regions of enrichment or depletion while both *syp-1* and *spo-11; syp-1* mutants exhibit dispersed H3K9me2. Scale bar represents 4 microns.

We monitored synapsis checkpoint activation in *mes-4 syp-1* double mutants. In *syp-1* mutant animals, the SC fails to assemble between all six pairs of homologs [28] and both the DNA damage and synapsis checkpoints are activated, resulting in very high levels of apoptosis (Figure 4A and [4]). When we assayed apoptosis in *mes-4 syp-1* double mutants, we did not see a decrease in the levels of apoptosis (Figure 4A), indicating that when all six pairs of homologous chromosomes are unsynapsed, *mes-4* is not required for the checkpoint.

Most reported examples of heterochromatin assembly on unsynapsed chromosomes in *C. elegans* involve situations in which there is partial asynapsis [8], [15]–[19]. If complete asynapsis does not produce robust heterochromatin assembly on all unsynapsed chromosomes, potentially leaving some chromosomal loci, including PCs, euchromatic by default, this might explain why *mes-4* is dispensable for checkpoint activation in *syp-1* mutants. To address this concern, we visualized H3K9me2 staining in *syp-1* mutants. In contrast to wildtype animals, in which meiotic chromosomes exhibited areas of enrichment or depletion of H3K9me2 (Figure 1C and 1D, Figure 4B and 4C), *syp-1* mutants exhibited dispersed H3K9me2 that colocalized with all meiotic chromosomes (Figure 4D and 4E), indicating that when all chromosomes are unsynapsed, they become enriched for H3K9me2. This has been reported in *syp-2* mutants [40], which are also defective in SC formation [41]. Since unsynapsed chromosomes undergo heterochromatin assembly in *syp-1* mutants (Figure 4D and 4E), we cannot attribute our finding that *mes-4* function is dispensable for synapsis checkpoint activation in this mutant background to differences in H3K9me2 enrichment when all chromosomes are unsynapsed.

We reasoned that *mes-4*'s role in the synapsis checkpoint may be redundant with another methyltransferase when all six pairs of chromosomes are unsynapsed. In both germlines and embryos, both *mes-4* and *met-1* are required to maintain wildtype levels of H3K36me3 (T. Takasaki and S. Strome, personal communication and [20]). To test whether *met-1* is required for the synapsis checkpoint in *syp-1* mutants, we knocked down *met-1* in *mes-4 syp-1* double mutants by RNA interference (*met-1; mes-4* double mutants exhibit defects in germline organization making analysis of apoptosis problematic [data not shown]). *mes-4* mutants can be resistant to RNAi but this resistance is dosage dependent [42]. RNAi (Figure 4A) or mutation (data not shown) of *met-1* in wildtype and *syp-1* single mutants did not reduce germline apoptosis in either genotype, indicating that loss of *met-1* alone behaves like loss of *mes-4* alone and does not affect physiological apoptosis or abrogate the synapsis checkpoint (Figure 4A). When we assayed germline apoptosis in *mes-4 syp-1* mutant hermaphrodites in which *met-1* had been knocked down by RNAi, we observed a decrease in apoptosis (Figure 4A). When compared to apoptosis in *mes-4 syp-1* double mutants, this decrease was statistically significant (p value = 0.0002) and similar to the levels of apoptosis observed when other components of the synapsis checkpoint, such as *pch-2* [4], are knocked down by RNAi (data not shown).

*spo-11; syp-1* double mutants only activate the synapsis checkpoint since the absence of SPO-11 induced double strand breaks in this mutant background prevents DNA damage checkpoint activation [4]. The absence of double strand breaks has no effect on heterochromatin assembly on unsynapsed chromosomes in *spo-11*; *syp-1* mutants (Figure 4F and 4G), indicating that H3K9me2 deposition occurs on unsynapsed chromosomes when the DNA damage checkpoint is not activated. When we assayed germline apoptosis in both *spo-11; mes-4 syp-1* triple mutants and *spo-11; syp-1* double mutants in which *met-1* had been knocked down by RNAi, we observed the same levels of apoptosis as observed in *spo-11; syp-1* double mutants (Figure 4A). However, when we knocked down *met-1* by RNAi in *spo-11; mes-4 syp-1* triple mutants, we saw a decrease in apoptosis to wildtype levels when compared to *spo-11; mes-4 syp-1* (p<0.0001) (Figure 4), indicating a loss of the synapsis checkpoint. To verify that the decrease in apoptosis we observed in *spo-11; mes-4 syp-1* triple mutants in which *met-1* had been knocked down by RNAi was due to abrogation of the synapsis checkpoint, we also assayed apoptosis in *pch-2; mes-4 syp-1* triple mutants in which *met-1* had been knocked down by RNAi and did not observe any additional decrease in the level of germline apoptosis (Figure 4A). These experiments indicate that *mes-4* and *met-1* act redundantly in the synapsis checkpoint when all chromosomes are unsynapsed.

### MET-1 is not required for the synapsis checkpoint when only *X* chromosomes are unsynapsed

**Figure 5:**
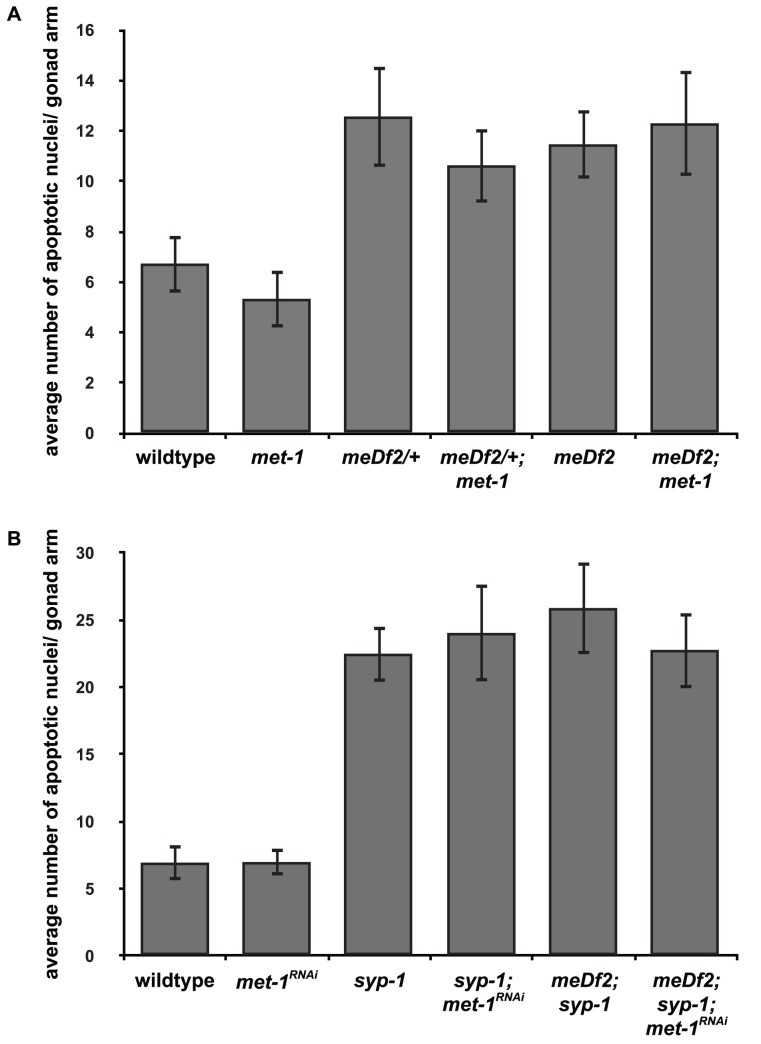
*met-1* is not required for the synapsis checkpoint when only *X* chromosomes are unsynapsed. A. Mutation of *met-1* does not reduce apoptosis in *meDf2/+* or *meDf2* homozygotes. B. Inactivation of *met-1* by RNAi in *meDf2; syp-1* double mutants does not reduce apoptosis. Error bars indicate 2XSEM.

We hypothesized that the requirement for *mes-4* and *met-1* activity for synapsis checkpoint activation in *syp-1* mutants might be explained by a model in which MES-4's role in the checkpoint is specific to *X* chromosomes and MET-1's role is specific for autosomes. To test this model, we performed two experiments. First, we monitored apoptosis in *meDf2* heterozygotes in which *met-1* had been mutated (*met-1; meDf2/+*). We found that apoptosis remained as high in *met-1; meDf2/+* double mutants as in *meDf2/+* single mutants (Figure 5A) (p value = 0.125). Thus, *met-1* is not required for the synapsis checkpoint when only *X* chromosomes are unsynapsed. We also determined that *met-1* is not required for the DNA damage checkpoint since apoptosis in both *met-1; meDf2* double mutants and *meDf2* single mutants was similar (Figure 5A).

Having demonstrated that *met-1* is not required to monitor synapsis of *X* chromosomes, we also tested whether *met-1* is uniquely required to monitor synapsis of autosomes. We previously showed that autosomes activate the synapsis checkpoint by introducing the *meDf2* deficiency into *syp-1* mutants [4]. In this double mutant background, only autosomes are capable of activating the synapsis checkpoint, since the absence of an active PC prevents *X* chromosomes from activating the synapsis checkpoint. We knocked down *met-1* gene function by RNAi in *syp-1; meDf2* double mutants and observed no effect on the levels of apoptosis (Figure 5B), similar to our results in *syp-1* single mutants (Figure 4A), indicating that *met-1* activity is not required to monitor synapsis of autosomes, likely because of the presence of *mes-4* (Figure 4A).

### MES-4's role in the synapsis checkpoint does not depend on heterochromatin assembly on unsynapsed *X* chromosomes

The requirement for *mes-4* function in the synapsis checkpoint combined with our observation that duplications with PC activity undergo heterochromatin assembly when *mes-4* is inactivated raises the possibility that MES-4's primary role in the checkpoint is to antagonize H3K9me2 enrichment at *X* chromosome PCs when they are unsynapsed. A similar role has recently been attributed to MES-4 in the context of MES-2/MES-3/MES-6 mediated H3K27me [34]. To test this model, we monitored synapsis checkpoint activation in hermaphrodites in which both *mes-4* and *met-2* gene functions were knocked down. If *mes-4*'s role in the checkpoint is dependent on *met-2* function, this result would support a model in which the primary role of MES-4 (and by extension MET-1) in the synapsis checkpoint is to prevent heterochromatin assembly at PCs. If *mes-4* is required for the synapsis checkpoint when *met-2* function and heterochromatin assembly on unsynapsed chromosomes is reduced, this might suggest that MES-4 (and by extension MET-1) is performing some other function at PCs and this indirectly prevents H3K9me2 enrichment at PCs.

**Figure 6:**
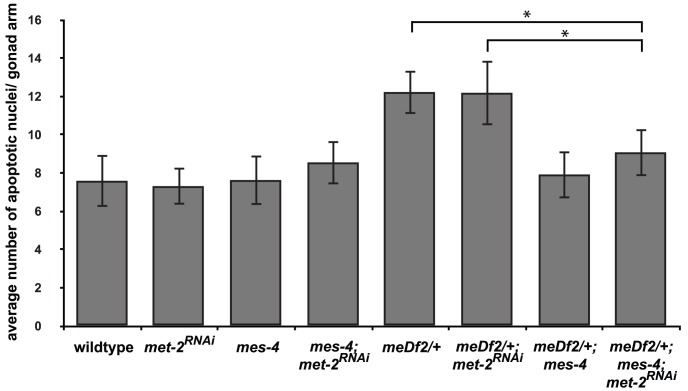
*mes-4*'s role in the synapsis checkpoint is independent of *met-2* mediated heterochromatin assembly on unsynapsed X chromosomes. *meDf2/+; mes-4* double mutants have nearly wild-type levels of apoptosis when *met-2* is inactivated by RNA interference. A * indicates a p value of <0.05. Error bars indicate 2XSEM.

We reduced H3K9me2 by RNAi of *met-2* in *meDf2/+; mes-4* mutants [17]. We verified that *met-2* had been sufficiently knocked down by monitoring the loss of H3K9me2 by immunofluorescence (data not shown). When *met-2* is knocked down by RNAi in wildtype and *meDf2/+* hermaphrodites, we did not observe a decrease in apoptosis, indicating that *met-2* is not required for physiological apoptosis or activation of the synapsis checkpoint (Figure 6). Similar results were obtained when *met-2* was inactivated by RNAi in *meDf2* homozygotes (data not shown), consistent with other reports that *met-2* is not required for the DNA damage checkpoint [15]. When *met-2* was knocked down by RNAi in *mes-4*; *meDf2/+* double mutants, the level of apoptosis was similar to what was observed in *mes-4*; *meDf2/+* double mutants (p value = 0.20) and significantly lower than what is observed in *meDf2/+* mutants (p value = 0.0005) and *meDf2/+* mutants in which *met-2* had been inactivated by RNAi (p value = 0.005) (Figure 6). These data argue that MES-4's role in the synapsis checkpoint does not necessarily depend on heterochromatin assembly on unsynapsed chromosomes. In the absence of *met-2* and detectable H3K9me2 on unsynapsed chromosomes, the synapsis checkpoint still requires MES-4 activity, presumably at PCs.

Heterochromatin assembly in the hermaphrodite germline appears to be the consequence of several pathways [17]–[19]. For example, *sin-3* activity has been shown to be required for heterochromatin assembly in the hermaphrodite germline but not for MSCI in the male germline [15]. *sin-3; met-2* double mutants are sterile [15], preventing us from using the double mutant to downregulate both pathways. We tested whether *mes-4* activity was required for the synapsis checkpoint when heterochromatin assembly on unsynapsed chromosomes was compromised by a reduction in *sin-3* gene function but observed that *mes-4* mutant hermaphrodites exposed to *sin-3* RNAi were sterile a generation earlier than *mes-4* single mutants (data not shown), precluding our ability to perform this experiment as well. This genetic interaction has also been observed when other chromatin modifiers, such as *set-2*, are depleted in *mes-4* hermaphrodites [43]. In light of these results, we cannot completely rule out the possibility that the requirement for *mes-4* in synapsis checkpoint activation in the absence of *met-2* reveals that other pathways that regulate heterochromatin assembly on unsynapsed chromosomes are still active. However, the absence of detectable H3K9me2 in *mes-4*; *meDf2/+* animals in which *met-2* had been inactivated by RNAi leads us to favor the model that *mes-4*'s role in the checkpoint does not necessarily depend on heterochromatin assembly on unsynapsed chromosomes.

### Is H3K36me3 the relevant chromatin modification for synapsis checkpoint activation?

**Figure 7:**
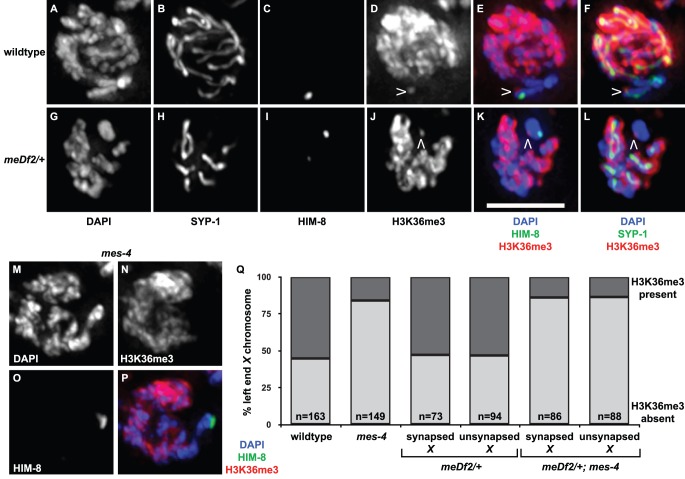
Is H3K36me3 the relevant chromatin modification for synapsis checkpoint activation? A–L. Indirect immunofluorescence was performed on meiotic nuclei in wildtype and *meDf2* heterozygotes against SYP-1, HIM-8 and H3K36me3. H3K36me3 is depleted from *X* chromosomes in both of these genetic backgrounds except for a single dot near the end (carets in D, E, F, J, K and L) that does not colocalize with HIM-8 (E and K). Scale bar represents 4 microns. M–P. Mutation of *mes-4* results in a reduction of H3K36me3 at the left end of *X* chromosomes. Indirect immunofluorescence was performed on meiotic nuclei in *mes-4* single mutants against HIM-8 and H3K36me3. Q. Quantification of the percentage of meiotic nuclei in wildtype, *mes-4* mutants, *meDf2* heterozygotes and *meDf2/+; mes-4* double mutants with H3K36me3 at the left end of X chromosomes. Mutation of *mes-4* reduces but does not eliminate the percentage of *X* chromosomes that exhibit this chromatin modification. *X* chromosomes in *meDf2* heterozygotes, whether synapsed or unsynapsed, exhibit this chromatin modification with a frequency similar to that observed in wildtype animals.

Mutation of *mes-4* eliminates detectable H3K36me2 [33] and reduces H3K36me3 in the germline; mutation of both *mes-4* and *met-1* is required to lose detectable H3K36me3 in the germline (T. Takasaki and S. Strome, personal communication). Since *mes-4* and *met-1* are redundant for the synapsis checkpoint when autosomes are unsynapsed, our data suggest that H3K36me, in particular H3K36me3, may be an important histone modification for signaling events at the PCs of unsynapsed chromosomes. To test this possibility, we performed indirect immunofluorescence against H3K36me3 in wildtype and *meDf2* heterozygotes (Figure 7A–7L). We observed visible enrichment of H3K36me3 at PC ends of *X* chromosomes in mid-pachytene meiotic nuclei in wildtype hermaphrodites, even as the rest of the chromosome appears depleted of this methyl mark (Figure 7D–7F), as has been reported [44]. When we quantified the percentage of mid-pachytene nuclei that exhibited H3K36me3 at the left end of the *X* chromosome, we determined that 55% of *X* chromosomes exhibited this chromatin mark in wildtype meiotic nuclei (Figure 7Q). We performed the same experiment in *meDf2* heterozygotes and observed that a very similar percentage of *X* chromosomes exhibited H3K36me3 at the left end of X chromosomes (Figure 7Q), whether synapsed (53%) or unsynapsed (53%) (Figure 7J–7L). The slight difference in the size of this mark presented in the wildtype and *meDf2* heterozygote images is not typical (data not shown). Furthermore, despite its localization to the left, or PC, end of *X* chromosomes [44], H3K36me3 did not colocalize with HIM-8 (Figure 7E and 7K) and is not present on duplications that contain PC activity (data not shown). We also found that enrichment of H3K36me3 at the left end of *X* chromosomes [44] is not dependent on HIM-8 function (data not shown), which is required for the ability of *X* chromosome PCs to activate the synapsis checkpoint [4].

We assessed whether the appearance of H3K36me3 at the left end of the *X* chromosome was dependent on *mes-4* (Figure 7M–7P). The majority of meiotic nuclei in mid-pachytene did not exhibit H3K36me3 at the left end of the X chromosome in the absence of *mes-4* (86%) (Figure 7M–7Q). A small fraction of meiotic nuclei did exhibit H3K36me3 (14%) in *mes-4* mutant animals (Figure 7Q), suggesting that *met-1* contributes to H3K36me3 at this locus. We observe a similar reduction in the percentage of meiotic nuclei that exhibit H3K36me3 at the left end of *X* chromosomes in *met-1* mutants (data not shown). The percentage of *X* chromosomes with H3K36me3 at the left end in *mes-4*; *meDf2/+* double mutants was similar to *mes-4* single mutants, whether *X* chromosomes were synapsed or unsynapsed (Figure 7Q). Taken all together, these results raise the intriguing possibility that H3K36me3 may not be the relevant MES-4/MET-1 catalyzed chromatin modification for synapsis checkpoint activation (see Discussion).

We also monitored H3K36me3 at the PC end of *X* chromosomes in *mes-4* mutant males and *met-1* mutant males to determine the genetic requirements for this specific methyl mark in males. Unlike hermaphrodites (Figure 7N, 7P and data not shown), we observed that H3K36me3 at the PC end of *X* chromosomes was completely absent in both *mes-4* mutant males and *met-1* mutant males (data not shown).

## Discussion

Our data suggest that chromatin state is an important aspect of the ability of PCs to activate the synapsis checkpoint. A chromosomal duplication that is sufficient to activate the synapsis checkpoint is less likely to undergo heterochromatin assembly, specifically enrichment of H3K9me2 (Figure 2D and 2G, Figure 3E). This distinguishes this duplication from duplications without PC activity, which consistently become enriched for H3K9me2 (Figure 2E and [8], [9], [19]). Furthermore, inactivation of a histone-modifying enzyme associated with active transcription and required for the synapsis checkpoint, MES-4 (Figure 3A), eliminates this distinction, producing a duplication with PC activity that undergoes heterochromatin assembly (Figure 3D and 3E). Our results lead us to propose that MES-4 and MET-1 generate a transcriptionally permissive chromatin environment at PCs that is required for synapsis checkpoint activation. We interpret the absence of H3K9me2 at PCs as an indirect result of MES-4 or MET-1 activity at PCs.

Given the difference in size between *mnDp3* (∼5 Mb) and *mnDp73* (∼2 Mb) [29], [30, Wormbase], our inability to observe enrichment of H3K9me2 on *mnDp73* may reflect a significantly weaker fluorescence signal of the antibody against H3K9me2 on *mnDp73*, which could be below the detection limit. However, when *mes-4* is inactivated by RNAi, *mnDp73* is visibly enriched with H3K9me2 (Figure 3D and 3E) in 88% of meiotic nuclei in which the duplication can be identified by HIM-8 recruitment, indicating that we can reproducibly observe heterochromatin assembly on this smaller duplication with cytological techniques.

It is unlikely that our results could be explained by mutation of *mes-4* and/or *met-1* disrupting transcription of another locus (or loci) important for checkpoint function: if *mes-4* and/or *met-1* were regulating the transcription of some other checkpoint component(s), we would not expect that asynapsis of the *X* chromosome would exhibit different genetic requirements for checkpoint activation than asynapsis of autosomes. Of the multiple checkpoint components identified thus far, both published [4], [38] and unpublished (data not shown), *mes-4* and *met-1* are the only components that exhibit chromosome specific effects.

### A role for transcription at PCs during synapsis checkpoint activation?

Both MES-4 and MET-1 are associated with active transcription: in embryos, MES-4 binds genes that were expressed in the maternal germline, potentially as a mechanism to transmit the epigenetic memory of maternal germline gene expression, while MET-1 is responsible for the deposition of transcription coupled H3K36me [20]. In the adult germline, loss of *mes-4* leads to an altered distribution of MES-2/MES-3/MES-6 repressive activity on chromatin, resulting in upregulation of genes on *X* chromosomes and autosomal genes whose expression is normally restricted to somatic tissue [33], [34]. *mes-4* mutant germ cells also exhibit downregulation of autosomal genes whose expression is normally enriched in or restricted to the germline, consistent with a role for MES-4 in promoting germline transcription. This role in promoting proper germline gene expression may be independent of the MES-2/MES-3/MES-6 complex, since *mes-2; mes-4* double mutants do not alleviate the downregulation of autosomal genes, as might be expected if MES-4's only role is to antagonize the repressive activity of the MES-2/MES-3/MES-6 complex [34]. Since we observe that *mes-4*'s role in the synapsis checkpoint is independent of the MES-2/MES-3/MES-6 complex (Figure 3B), we favor a scenario in which MES-4 (and MET-1) activity directly contributes to transcriptional activity at PCs to promote checkpoint activation.

This hypothesis is supported by our finding that *mes-4* is still required for synapsis checkpoint activation in the absence of *met-2* mediated heterochromatin assembly on unsynapsed chromosomes (Figure 6). Since apoptosis in response to synapsis checkpoint activation is transcriptionally regulated (J.M. Ragle and N. Bhalla, unpublished observations), we cannot directly test whether transcription is required for synapsis checkpoint activation, for example, by RNAi depletion of AMA-1, the large subunit of RNA Polymerase II [33]. We addressed this issue cytologically by using an antibody against the serine 5 phospho-epitope of RNA Polymerase II that is associated with transcriptional competence [45] and did not observe any enrichment of this specifically modified version of RNA Polymerase II on *mnDp73* (data not shown). However, the observed inconsistency between cytological (reviewed in [46]) and more refined assays of transcriptional activity in the germline [20], [47], [48] raises the possibility that cytological approaches may not be adequately informative.

It is possible that MES-4 and MET-1 may not be mediating transcriptional activity at PCs. This would be consistent with our inability to localize modified RNA Polymerase II on *mnDp73* (data not shown). While MES-4 and MET-1 mediated catalysis of H3K36me is associated with active transcription [20], studies in budding yeast have shown that Set2, the enzyme that methylates H3K36 in response to transcription, is responsible for creating a repressive transcriptional environment within the body of an actively transcribed gene to prevent inappropriate, or cryptic, transcriptional activation [23], [24], [25]. While we favor the interpretation that the requirement for MES-4 and MET-1 in synapsis checkpoint activation suggests a role for active transcription at PCs, we cannot rule out that MES-4 and MET-1 may contribute to a repressive chromatin environment at PCs that is distinct from the enrichment of H3K9me2 that is observed on the bulk of unsynapsed chromosomes. Additional studies will dissect the precise roles of MES-4 and MET-1 in the synapsis checkpoint.

Identifying the loci that may be transcribed to promote checkpoint activation is an obvious focus of our future investigations. Each PC is enriched with repetitive sequence elements [49] and it is tempting to speculate that transcription of these repetitive elements contributes to checkpoint activation. We tested whether extrachromosomal arrays composed of these repeats could activate the checkpoint but did not observe synapsis checkpoint activation in array-bearing hermaphrodites (data not shown). Although these arrays are competent for PC protein recruitment, pairing and synapsis and do not appear to undergo H3K9 methylation (data not shown), a chromosomal context or other sequence elements, such as promoters and enhancers, may be necessary for transcription and synapsis checkpoint activation.

To our knowledge, no data currently suggests that transcription is required for the other characterized role that PCs play in mediating pairing and synapsis [5]–[7], [38], [50]–[52]. *mes-4* mutant homozygotes from heterozygote mothers are fertile (as a result of the maternal contribution of MES-4 that supports germline development) and don't exhibit a Him (high incidence of male) phenotype [21], indicating that *mes-4* is not required for *X* chromosome segregation during meiosis. *met-1; mes-4* double mutants exhibit low brood size, high embryonic lethality and some degree of larval arrest but no obvious Him phenotype among the sterile survivors (T. Takasaki and S. Strome, personal communication), suggesting that meiotic defects are not the cause of the embryonic lethality. One possible model is that transcription of loci at PCs is only required for the checkpoint. The ability to uncouple pairing and synapsis from checkpoint activation with arrays that recruit PC proteins might suggest that this is true. Alternatively, factors in addition to MES-4 and MET-1 may regulate transcription at PCs and be required for pairing and synapsis.

Four paralogous zinc-finger proteins (HIM-8, ZIM-1, ZIM-2, and ZIM-3) are required for PC function [6], [7]. PC proteins have been implicated in regulating transcription in somatic cells, independent of their roles in meiosis [53], [54]. In a variety of somatic contexts, mutations in *him-8* and the *zim* genes suppress mutant phenotypes that arise from hypomorphic mutations in transcription factors [54]. Interestingly, this role in transcriptional regulation is strictly dependent on the ability of PC proteins to bind DNA since an allele of *him-8 (me4)* that supports DNA binding but not *X* chromosome pairing or synapsis doesn't exhibit this genetic interaction [53]. In the context of the synapsis checkpoint, the *him-8(me4)* allele is as defective as other alleles of *him-8* (*mn253* and *e1489*) tested for a role in the checkpoint (data not shown and [4]), indicating that any contribution to transcription at the PCs during checkpoint activation is likely to require all functions of HIM-8, ZIM-1, -2 and -3.

PCs have been compared to centromeres in monocentric organisms [50], [55]. In particular, it has been suggested that investing a single chromosomal locus with the ability to promote pairing and synapsis in the meiotic germline may contribute to genomic integrity in an organism, like *C. elegans*, in which chromosomes are holocentric and any deleterious changes to genome organization (i.e. the creation of translocations, rearrangements or chromosome fragments) will be faithfully transmitted during mitosis [55]. The use of PCs as sites of checkpoint activation extends this analogy since the centromere also monitors the event it engages in, namely spindle attachment. In a variety of monocentric organisms, transcription is required at centromeres for full activity and the faithful transmission of chromosomes [56]–[59]. A well-characterized contribution of transcription to centromere function involves the use of the RNAi machinery to establish and maintain pericentric heterochromatin [58]–[60]. However, these findings do not provide a satisfying framework to imagine how transcription may be contributing to PC function since the end product is silenced chromatin.

More recently, however, euchromatic domains, as defined by histone modifications associated with transcriptionally permissive chromatin, have been observed in core centromeric regions in rice [61], fruit flies, humans [62] and on human artificial chromosomes [63], suggesting that transcriptional activity aside from the formation of pericentric heterochromatin by the RNAi machinery may be required for aspects of centromere function. In some organisms, such as fission yeast, maize and humans, transcription of core centromeric regions has even been observed [64], [65], [66] and associated with centromere activity [57], [64], [67]. In budding yeast, a model organism that does not contain the RNA interference machinery, loss of transcription across centromeres results in chromosomal instability and sensitivity to microtubule depolymerizing drugs [56], phenotypic hallmarks of spindle assembly checkpoint mutants that fail to arrest mitosis in response to defects in spindle attachment [68].

### What chromatin modification is critical for synapsis checkpoint activation?

Both MES-4 and MET-1 are required for all detectable H3K36me3 in germline nuclei (T. Takasaki and S. Strome, personal communication), suggesting that this histone modification is potentially relevant for synapsis checkpoint activation. Indeed, we observe a reduction in H3K36me3 at the left end of *X* chromosomes in *mes-4* mutants (Figure 7N, 7P and 7Q). However, some of our data is inconsistent with H3K36me3 being the relevant MES-4/MET-1 catalyzed chromatin modification for synapsis checkpoint activation. Unsynapsed *X* chromosomes in *meDf2* heterozygotes exhibit similar levels of H3K36me3 enrichment at their PC end as synapsed chromosomes in *meDf2* heterozygotes or wildtype animals (Figure 7Q). Furthermore, loss of *met-1* function also results in a reduction of this signal at the PC end of *X* chromosomes (data not shown), even though *met-1* gene function is not required to monitor synapsis of *X* chromosomes (Figure 5A).

Other reports support the possibility that H3K36me3 is not critical for synapsis checkpoint activation. Knockdown of a characterized H3K36 demethylase (JMJD2) results in ectopic accumulation of H3K36me3 on the PC end of *X* chromosomes and DNA damage checkpoint activation in the germline but has no effect on synapsis checkpoint activation [44], indicating that enrichment of H3K36me3 at the left end of the X chromosome is not sufficient to activate the synapsis checkpoint. Moreover, the chromodomain protein identified as the putative H3K36me3 reader in *C. elegans*, MRG-1 [69], exhibits defects in synapsis [70] and activates the synapsis checkpoint when mutated [40], indicating that it is not required for the synapsis checkpoint.

Mutation of *mes-4* also results in loss of detectable H3K36me2 in germline nuclei, suggesting it is the sole histone methyltransferase responsible for this methyl mark. However, given the potential inconsistencies between cytological and more refined assays to monitor chromatin modifications (see above), this does not necessarily mean that MET-1 cannot contribute to this mark. Therefore, H3K36me2 remains a potential candidate chromatin modification critical for synapsis checkpoint activation.

Another possibility is that MES-4 and MET-1 activity may be required for the catalysis of a histone modification aside from H3K36me to promote synapsis checkpoint signaling. For example, MES-2 was known to be required for H3K27me2 and H3K27me3 in the germline [36] but recent studies have also revealed that it is responsible for most of the detectable H3K9me3 as well [17]. One candidate histone modification that may be linked to synapsis checkpoint activation is H3K4me. This histone mark, which is associated with active transcription [71], has been observed on sDp1 [8], a large autosomal duplication that has PC activity [72]. Given that we suspect that PCs need to maintain transcriptional activity to monitor synapsis, this histone modification may maintain an active transcriptional state by recruiting transcriptional activators or it may specifically recruit proteins that recognize this methyl mark (e.g. a chromodomain protein) in checkpoint activating strains to promote checkpoint signaling. Future experiments will distinguish between these two models. An alternate hypothesis is that MES-4 and MET-1 are methylating something instead of histones at PCs to promote checkpoint signaling [73].

### Why are there different mechanisms to monitor synapsis of the *X* chromosome and autosomes?

We have shown that *X* chromosomes require *mes-4* to monitor synapsis while *mes-4* and *met-1* are redundant to monitor autosomal asynapsis. The *X* chromosome in *C. elegans* is distinct from the autosomes in several respects: it exhibits a more uniform distribution of gene density, recombination rates and repeat content [74]; it expresses genes in the germline [47], [48] at a reduced level [48], potentially as a result of histone modifications associated with transcriptional silencing (reviewed in [46]); it responds differently to hypomorphic mutation of certain genes required for meiosis [75]–[77]; it is the target of dosage compensation during sex determination and development [78]; and during male meiosis the single *X* chromosome segregates without a pairing and recombination partner.

We speculate that the unique requirement of the *X* for *mes-4* activity in the synapsis checkpoint is a consequence of the male genotype (*XO*). When all germline nuclei have an unsynapsed sex chromosome, as they do in males, having distinct requirements for checkpoint activation for sex chromosomes and autosomes may allow the checkpoint to be unresponsive to the presence of an unsynapsed *X* chromosome while still maintaining the ability to monitor the synapsed state of the autosomes. Unsynapsed *X* chromosomes in males are specifically shielded from activating the DNA damage checkpoint in the germline by *met-2* mediated MSCI [15]. However, loss of MSCI does not produce synapsis checkpoint activation [15] despite the proper localization of HIM-8 to the unsynapsed *X* chromosomes [7], indicating that some other mechanism prevents synapsis checkpoint activation in male germlines. To address whether male germlines are competent to activate the DNA damage checkpoint despite the absence of apoptosis [79], Engebrecht and colleagues have taken advantage of cytological markers of DNA damage checkpoint activation to elegantly illustrate that this checkpoint can be fully activated in males and contributes to genomic integrity [80]. Unfortunately, we currently have no comparable cytological markers of synapsis checkpoint activation and therefore can only assess checkpoint activation by monitoring apoptosis. The development of reagents that allow us to assess synapsis checkpoint activation independent of apoptosis will allow us to directly address how the unsynapsed male *X* is shielded from synapsis checkpoint activation.

Our model that *mes-4*'s unique role in monitoring synapsis of *X* chromosomes provides a mechanism for the observed sexual dimorphism in checkpoint activation predicts that *mes-4* activity is either inhibited or unnecessary in male germlines. It is currently unclear whether *mes-4* is required in males for germline development (Gaydos, L. and S. Strome, personal communication). It is possible that the difference we observe between male and hermaphrodite germlines in the genetic requirements for H3K6me3 at the PC end of *X* chromosomes (data not shown and Figure 7N and 7P) indicates that these genes play different roles depending on the sex of the germline. This interpretation would be consistent with a recent study that found that knockdown of *met-1* and *mes-4* by RNAi also resulted in an increase in germline apoptosis in genotypically male germlines [15].

## Materials and Methods

### Genetics

The wildtype *C. elegans* strain background was Bristol N2. All experiments were performed at 20° under standard conditions. Mutations and rearrangements used were as follows:

*LG I: mnDp66, met-1(n4337), mes-3(bn35)*

*LG II: mes-2(bn11), pch-2(tm1458)*

*LG III: met-2(n4256)*

*LG IV: spo-11(ok79), nT1[unc-?(n754) let-?(m435)] (IV, V)*

*LG V: dpy-11(e224)*, *mes-4(bn23)*, *syp-1(me17), bcIs39(P_lin-15_::ced-1::GFP)*

*LG X: unc-1(e1598n1201), dpy-3(e27), meDf2, mnDp73*, *unc-3(e151), mnDp3*

Experiments with *mes* mutants were performed in the M+Z- generation, in which maternal load of the relevant gene function allows the generation of a fertile homozygote adult with a developed germline.

Some nematode strains used in this work were provided by the Caenorhabditis Genetics Center, which is funded by the NIH National Center for Research Resources (NCRR).

### Scoring of apoptotic nuclei

Scoring of apoptotic nuclei was performed as in [4]. To assess significance, two-tailed unpaired t tests were performed to compare average number of apoptotic nuclei between genotypes.

### RNA interference

All RNAi was performed by culturing relevant worm strains on HT115 bacteria transformed with vectors that allowed for IPTG inducible expression of dsRNA. Bacteria containing RNAi vectors were grown overnight at 37°, centrifuged and resuspended in 1/20 the original volume. 50 ul of this concentrated culture was spotted onto NGM plates containing 1 mM IPTG and 50 ug/ul carbenicillin. After incubation at 37° overnight, late L4 stage worms were picked into M9, transferred to these plates and allowed to crawl in the bacteria for two hours to clear OP50 from their gut. They were then transferred to fresh RNAi plates, allowed to produce progeny and L4s from this population were picked to score apoptosis or perform cytology.

To reproducibly knock down *met-2* activity by feeding RNAi, we generated a new *met-2* RNAi vector using a Gateway-compatible (Invitrogen) RNAi vector that included termination sites downstream of the T7 promoters (pDONRT7) [81]. *met-2* was amplified by PCR from N2 genomic DNA using primers met-2_FOR (5′ ggg/gac/aag/ttt/tgt/aca/aaa/aag/cag/gct/gca/cca/atc/aga/atg/tcg 3′) and met-2_REV (5′ ggg/gac/cac/ttt/gta/caa/gaa/agc/tgg/gtt/ttt/cca/gct/cca/cgg/tat/c 3′). The resulting PCR product was cloned into pDONRT7 using a BP reaction (Invitrogen) and transformed into DH5α. A clone in which the *met-2* PCR product was properly inserted into pDONRT7 was identified, isolated and retransformed into HT115.

### Immunostaining and microscopy

Immunostaining was performed as in [4]. Primary antibodies were as follows (dilutions are indicated in parentheses): rabbit anti-SYP-1 (1∶250) [28], guinea pig anti-HTP-3 (1∶250) [7], guinea pig anti-HIM-8 (1∶250), guinea pig anti-SUN-1 pSer8 (1∶700), guinea pig anti-SUN-1 pSer12 (1∶17,000), mouse anti-H3K9me2 (1∶50,000) [82], mouse anti-H3K36me3 (1∶50,000) [20] and rabbit anti-H3K36me3 (Abcam). Secondary antibodies were Cy3 anti-mouse and anti-rabbit pig (Jackson Immunochemicals), Alexa-Fluor 555 anti-rabbit (Invitrogen) and Cy5 anti-guinea pig (Jackson Immunochemicals). We thank Anne Villeneuve, Abby Dernburg, Verena Jantsch and Hiroshi Kimura for reagents.

All images were acquired using a DeltaVision Personal DV system (Applied Precision) equipped with a 100× N.A. 1.40 oil-immersion objective (Olympus), resulting in an effective XY pixel spacing of 0.064 or 0.040 µm. Three-dimensional image stacks were collected at 0.2-µm Z-spacing and processed by constrained, iterative deconvolution. Image scaling and analysis were performed using functions in the softWoRx software package. Projections of complete meiotic nuclei were calculated by a maximum intensity algorithm. Composite images were assembled and some false coloring was performed with Adobe Photoshop. In some cases, projections of partial Z-stacks of meiotic nuclei were generated (for example, Figure 2E–2J, Figure 3C and 3D, and Figure 7G–7L) to facilitate the visualization of duplications or unsynapsed chromosomes. In Figure 2C, the non-PC region was identified as a region not adjacent to the HIM-8 signal and of similar size to observed instances when the PC region was devoid of H3K9me2.

## Supporting Information

Figure S1 H3K9me2 can be observed adjacent to HIM-8 on unsynapsed *X* chromosomes. Indirect immunofluorescence was performed on meiotic nuclei in *meDf2* heterozygotes using antibodies against the *X* chromosome PC protein HIM-8 and H3K9me2. Color and gray scale images (A–D) of two meiotic nuclei with unsynapsed *X* chromosomes (see Figure 2B). The nucleus on the left exhibits H3K9me2 enrichment adjacent to a HIM-8 signal, as outlined. Scale bar represents 4 microns.
(PDF)

Figure S2 *mnDp3* is often synapsed when it undergoes heterochromatin assembly. Indirect immunofluorescence was performed on meiotic nuclei in hermaphrodites carrying *mnDp3* using antibodies against the SC component SYP-1 (A and C) and H3K9me2 (A and B). Arrows indicate meiotic nuclei in which the duplication is the primary H3K9me2 signal in the nucleus and load SYP-1. This was observed in 37 of 60 (62%) meiotic nuclei in which a primary H3K9me2 signal indicated the presence of a duplication.
(PDF)

Figure S3 The delay in meiotic progression observed in *meDf2* heterozygotes is not affected by mutation of *mes-4*. Indirect immunofluorescence was performed against SUN-1 phosphorylated on serine 8 in germlines from wildtype hermaphrodites, *meDf2/+* mutant hermaphrodites and *meDf2/+; mes-4* double mutants.
(PDF)

Figure S4 The delay in meiotic progression observed in *meDf2* heterozygotes is not affected by mutation of *mes-4*. Indirect immunofluorescence was performed against SUN-1 phosphorylated on serine 12 in germlines from wildtype hermaphrodites, *meDf2/+* mutant hermaphrodites and *meDf2/+; mes-4* double mutants.
(PDF)
