# Supplementary figures and images for: Histone Methyltransferases MES-4 and MET-1 Promote Meiotic Checkpoint Activation in Caenorhabditis elegans
Source: PLoS Genet. 2012 Nov 15;8(11):e1003089. doi: 10.1371/journal.pgen.1003089 (PMC3499413; doi:10.1371/journal.pgen.1003089)

Supplemental Figure 1

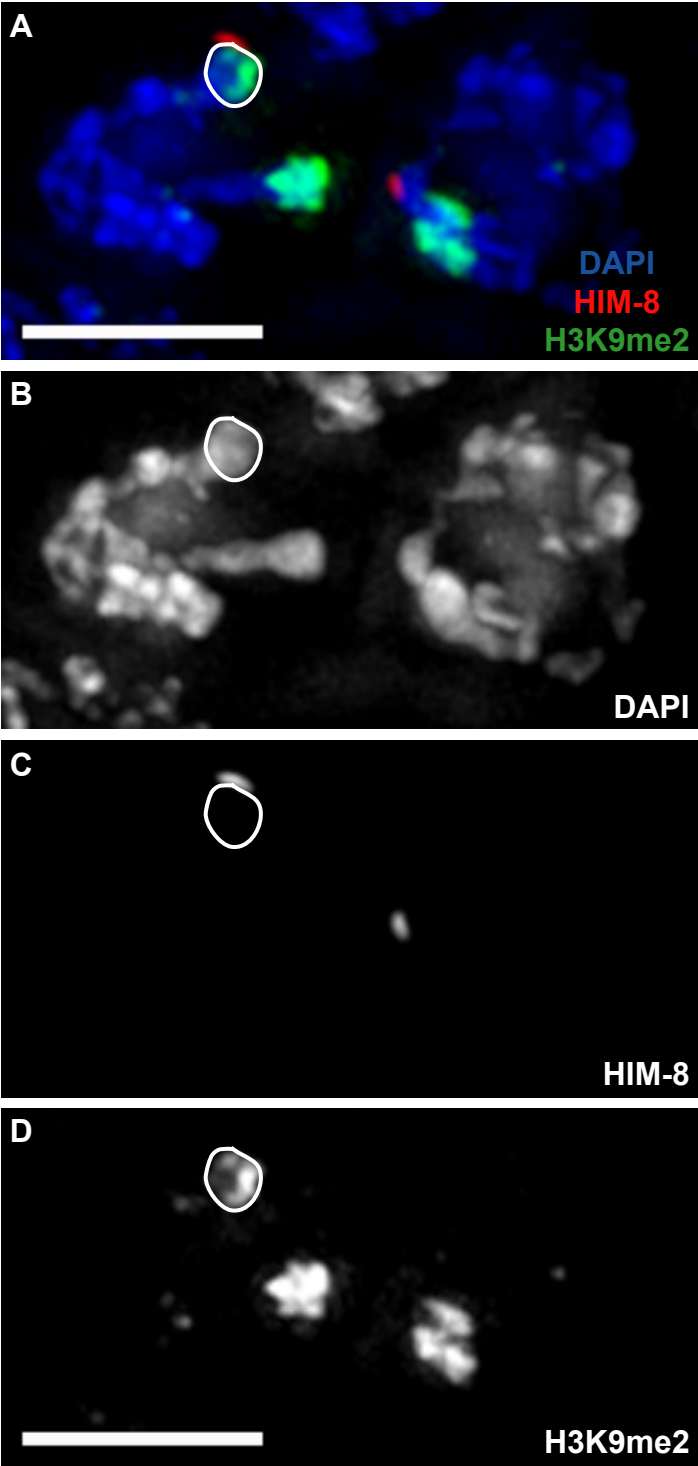

Supplement: Figure S1 — H3K9me2 can be observed adjacent to HIM-8 on unsynapsed X chromosomes. Indirect immunofluorescence was performed on meiotic nuclei in meDf2 heterozygotes using antibodies against the X chromosome PC protein HIM-8 and H3K9me2. Color and gray scale images (A–D) of two meiotic nuclei with unsynapsed X chromosomes (see Figure 2B). The nucleus on the left exhibits H3K9me2 enrichment adjacent to a HIM-8 signal, as outlined. Scale bar represents 4 microns. (PDF) [file pgen.1003089.s001.pdf]

Supplemental Figure 2

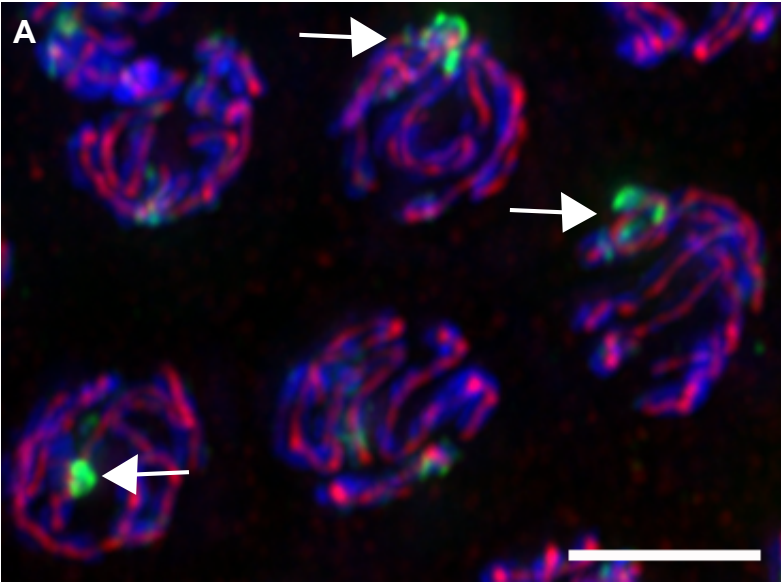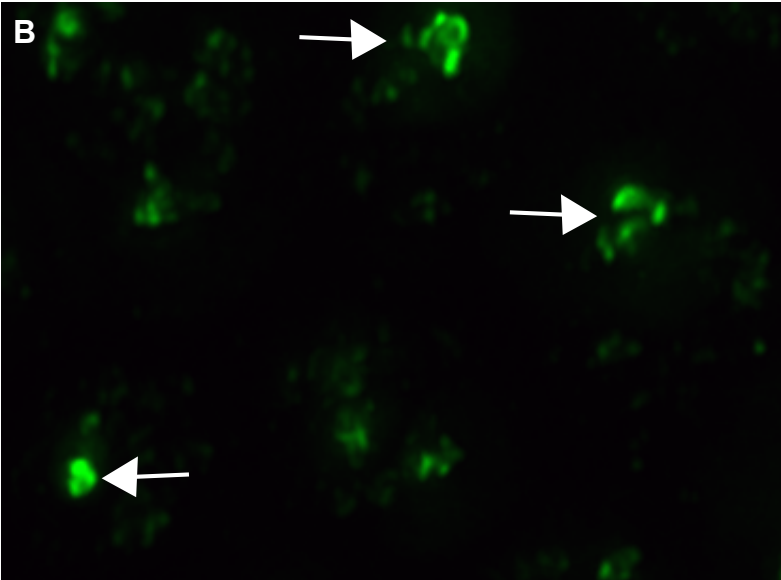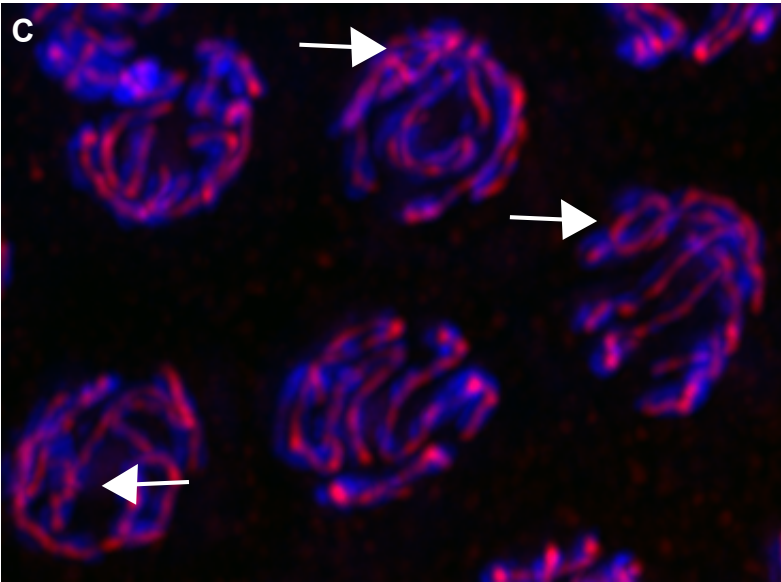

DAPI  
SYP-1  
H3K9me2

Supplement: Figure S2 — mnDp3 is often synapsed when it undergoes heterochromatin assembly. Indirect immunofluorescence was performed on meiotic nuclei in hermaphrodites carrying mnDp3 using antibodies against the SC component SYP-1 (A and C) and H3K9me2 (A and B). Arrows indicate meiotic nuclei in which the duplication is the primary H3K9me2 signal in the nucleus and load SYP-1. This was observed in 37 of 60 (62%) meiotic nuclei in which a primary H3K9me2 signal indicated the presence of a duplication. (PDF) [file pgen.1003089.s002.pdf]

Supplemental Figure 3

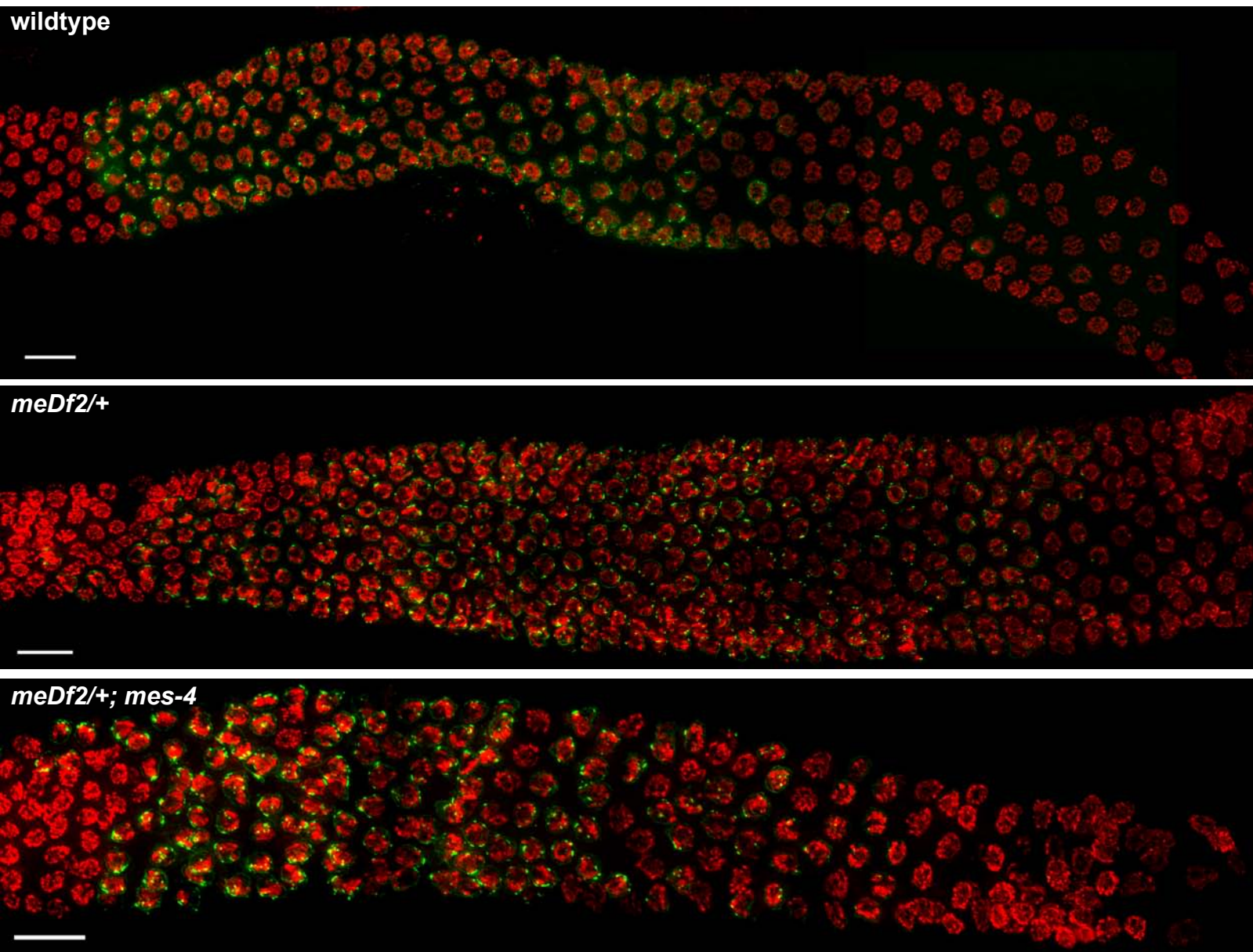

DAPI  
SUN-1 pSer8

Supplement: Figure S3 — The delay in meiotic progression observed in meDf2 heterozygotes is not affected by mutation of mes-4. Indirect immunofluorescence was performed against SUN-1 phosphorylated on serine 8 in germlines from wildtype hermaphrodites, meDf2/+ mutant hermaphrodites and meDf2/+; mes-4 double mutants. (PDF) [file pgen.1003089.s003.pdf]

Supplemental Figure 4

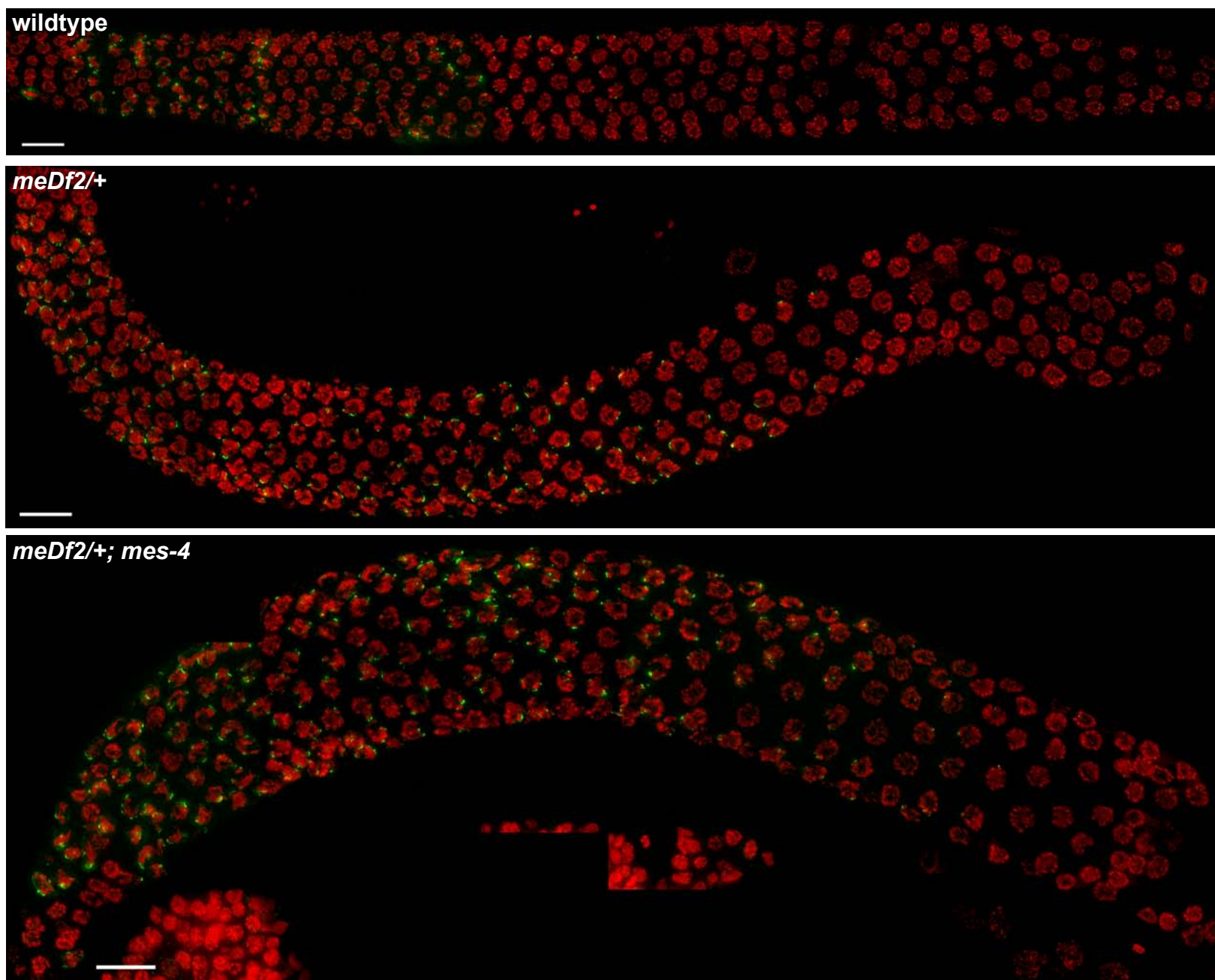

DAPI  
SUN-1 pSer12

Supplement: Figure S4 — The delay in meiotic progression observed in meDf2 heterozygotes is not affected by mutation of mes-4. Indirect immunofluorescence was performed against SUN-1 phosphorylated on serine 12 in germlines from wildtype hermaphrodites, meDf2/+ mutant hermaphrodites and meDf2/+; mes-4 double mutants. (PDF) [file pgen.1003089.s004.pdf]
